# A Microperfusion and In-Bore Oxygenator System Designed for Magnetic Resonance Microscopy Studies on Living Tissue Explants

**DOI:** 10.1038/srep18095

**Published:** 2015-12-15

**Authors:** Jeremy J. Flint, Kannan Menon, Brian Hansen, John Forder, Stephen J. Blackband

**Affiliations:** 1 Department of Neuroscience, University of Florida, Gainesville, Florida, United States of America; 2 McKnight Brain Institute, University of Florida, Gainesville, Florida, United States of America; 3 Department of Biomedical Engineering, University of Florida, Gainesville, Florida, United States of America; 4 Center for Functionally Integrative Neuroscience, Aarhus University, Aarhus, Denmark; 5 Department of Radiology, University of Florida, Gainesville, Florida, United States of America; 6 National High Magnetic Field Laboratory, Florida State University, Tallahassee, Florida, United States of America

## Abstract

Spectrometers now offer the field strengths necessary to visualize mammalian cells but were not designed to accommodate imaging of live tissues. As such, spectrometers pose significant challenges—the most evident of which are spatial limitations—to conducting experiments in living tissue. This limitation becomes problematic upon trying to employ commercial perfusion equipment which is bulky and—being designed almost exclusively for light microscopy or electrophysiology studies—seldom includes MR-compatibility as a design criterion. To overcome problems exclusive to ultra-high magnetic field environments with limited spatial access, we have designed microperfusion and in-bore oxygenation systems capable of interfacing with Bruker’s series of micro surface-coils. These devices are designed for supporting cellular resolution imaging in MR studies of excised, living tissue. The combined system allows for precise control of both dissolved gas and pH levels in the perfusate thus demonstrating applicability for a wide range of tissue types. Its compactness, linear architecture, and MR-compatible material content are key design features intended to provide a versatile hardware interface compatible with any NMR spectrometer. Such attributes will ensure the microperfusion rig’s continued utility as it may be used with a multitude of contemporary NMR systems in addition to those which are currently in development.

There is a well described and established clinical phenomenon that correlates increasing efficacy of disease treatment with decreasing time interval between disease inception and treatment application^1^,^2^,^3^. Unfortunately, most contemporary diagnoses rely on patient self-reporting of disease symptomatology prior to clinicians rendering diagnosis and treatment. This often permits severely protracted time periods of asymptomatic disease development prior to patients’ receiving necessary treatments.

While biomarker assays promise to provide exquisitely sensitive screening tools capable of early disease detection, thus shortening the time interval between onset and treatment, such tools generally offer little information regarding the spatial characteristics of tissue pathology within the living organism. Accurate information regarding the spatial characteristics of diseased tissue is not trivial in the case of patient care as clinicians must be able to distinguish between healthy and unhealthy tissues for a variety of applications including diagnostic confirmation, post-treatment disease monitoring, and surgical planning for tumor excision^4^. Alternatively, diagnostic methods which can offer tissue-specific spatial information—such as molecular labeling following tissue biopsy—are often too limited in the scope of their spatial data, unamenable to repeated measures or when investigating certain tissues, and too often result in secondary infections which complicate or prevent recovery^5^. In recent years, a resurgence in secondary infections has occurred—especially in the case of prostate biopsy—presumably due to an ongoing decline in the efficacy of postoperative antibiotics^6^,^7^.

Clearly, clinical imaging methods must develop in conjunction with improvements made in molecular methods of disease detection if we are to realize the full potential of either methodology to improve patient health. Identifying the MR characteristics of both healthy and diseased tissues at the cellular level will provide insight into how such early stage—often asymptomatic—disease conditions affect MR signal and contrast characteristics. Thus, while tissue microstructure currently is—and may remain—inaccessible to direct observation in the clinic, possessing a thorough understanding of how specific disease states affect MR contrast parameters in the microenvironment will lead to an understanding of how pathological changes occurring at the microscopic level present themselves in clinical scans. Such cellular-resolution data (<10 μm isotropic) must be collected using ultra-high-field imaging equipment as these are the only systems capable of quantifying MR contrast parameters at the relevant physical scale. Hence, there is a current need for MR imaging systems capable of resolving mammalian cellular architecture for use in healthy and diseased tissue characterization studies.

Preliminary MR imaging studies of mammalian cellular structure have been conducted exclusively in fixed tissue samples due to the lengthy collection time needed to produce cellular-resolution scans^8^,^9^. While these experimental conditions were necessary to ensure sample stability over the course of long imaging experiments, such conditions are not ideal as fixative is known to alter tissues’ osmotic properties, membrane permeability, and relaxation characteristics^10^,^11^,^12^,^13^.

MR microscopy studies conducted on living tissue explants have traditionally suffered from low—i.e. non-cellular—resolution, as well as imprecise control of perfusate conditions at the site of the isolated tissue. The inability to directly visualize cellular structure has since been overcome through vast improvements in radio-frequency (RF) microcoil technology^14^,^15^,^16^,^17^,^18^,^19^,^20^,^21^,^22^. However, the ability to effectively control perfusate conditions during MR microimaging experiments has not been adequately addressed. Numerous iterations of perfusion devices are available for explant studies which offer superb control of sample conditions as well as innovative features, such as microneedle arrays, for improving metabolite delivery^23^,^24^,^25^,^26^. A key drawback of most contemporary perfusion systems however is the lack of MR compatibility during the concept and design stages of their development. As a result, modifying such devices so that they may be interfaced with existing MR imaging systems is highly impractical given both the spatial and material limitations imposed when working with ultra-high magnetic field scanners.

To address the need for reliable, MR compatible microperfusion hardware for use with living explants, we have designed and fabricated a tissue retention/perfusion system specifically for use in cellular-resolution studies. The prototype system interfaces to a modified, commercially available micro surface coil (Bruker Biospin, B6370). In addition to detailed schematics and fabrication procedures, we report the results of tests quantifying the dissolved oxygen content and pH of our artificial cerebrospinal fluid (aCSF) as well as MR signal stability over time in experiments where our in-bore oxygenator is in use. In the case of dissolved gas and pH quantification, results from tests using the in-bore oxygenator are compared to equivalent experiments employing an external membrane oxygenator device of earlier design^27^.

## Methods

### Design and Fabrication

#### Micro Surface-Coil Modification

A 500 μm diameter micro surface-coil (Bruker Biospin, Z76409) was modified to interface with the micro perfusion rig. A channel (15 mm LN × 3 mm HT × 4 mm D) was made in the back of the coil’s plastic assembly. Two nylon spacers (6 mm LN × 4 mm HT 0.5 mm W) were glued flanking the channel and filed down to the coil’s semicircular contour. Two narrower, bilateral groves (3 mm HT × 1.5 mm D) were extended from the channel until they wrapped around the coil assembly. A hole (2 mm Dia. × 14 mm D) was drilled in the top of the coil assembly centered along its width and located directly behind the chip plate.

#### Perfusion Rig Fabrication

High gas-retention perfusion lines (Cole-Parmer, 06508-13) were employed between the gassed perfusate reservoir and oxygenators. In the external oxygenaor setup, this line also bridged the oxygenator and perfusion well. A peristaltic pump (Masterflex L/S, 7519-20) drove perfusion (2 ml/min). For the perfusion chamber (150 μm volume), acetal rod was machined into a single, open-ended configuration (9.5 mm O.D., 6 mm LN, 6 mm I.D.). The open end was beveled 30^o^ to accommodate a silicone gasket (Amazon, ORS-009-25) that was adhered using silicone sealant. A horizontal channel (2.5 mm HT × 1 mm D) was milled into the perfusion well’s closed end to accommodate a cable tie (Thomas & Betts, SF100-18). Inflow and outflow lines (Cole-Parmer, S-06418-02) were held in place with urethane and were offset (3 mm) within the well’s interior to maximize turbulent flow and minimize metabolite gradients. Nylon retention ring (5 mm O.D., 4 mm I.D., 300 μm thick) and mesh (4 mm Dia., 2 mm × 1.5 mm window, 50 μm pore size) were fashioned by hand from a nylon washer (Amazon, B00DHVBPOO) and woven nylon sheet (Amazon, CMN-0053-C).

#### In-Bore Oxygenator Fabrication

A 5 mm diameter NMR tube (Wilmad, WG-1000-7) was modified into a glass pipe 16.5 cm in length to serve as the gas inlet (interior) chamber. A hole (1.5 mm Dia.) was cut in the top of the 5 mm tube cap to accommodate the carbogen supply line. Next, a 10 mm NMR sample tube (Wilmad, 513-1 PP-7FB) was modified into a glass pipe 18 cm long to form the gas exchange (exterior) chamber. A hole (6 mm Dia.) was cut in the center of the 10 mm tube’s cap to nest the 5 mm tube assembly. Two additional holes (1 mm Dia.) were made in the top of the 10 mm tube’s cap. One served as the exchange-membrane inlet while the other remained open to vent incoming carbogen. Silicone exchange tubing (HelixMark, 60-011-03) was introduced through the top 10 mm tube cap, coiled tightly around the nested 5 mm tube inside the gas-exchange compartment, and passed through a second 10 mm NMR tube cap located at the oxygenator’s base. This cap housed an acetal peg (20 mm HT, 2 mm Dia.) at its center held in place by urethane. Externalized lengths of gas exchange tubing were minimized to two 10.0 mm segments joined via a 1/16” nylon coupler (Eldon James Corp., C0-1 NN) to perfusion lines (Cole-Parmer, S-06418-02) offering slightly higher gas retention. The perfusion line exiting the bottom of oxygenator was coupled directly to the perfusion chamber’s inflow line. The lower 10 mm NMR tube cap was attached without urethane as a means to access the interior of the oxygenator device for membrane replacement.

### Characterization of Perfusate Conditions at the Tissue Well

#### Dissolved Oxygen Content

Measurements for percent dissolved oxygen of aCSF perfusate (120 mM NaCl, 26 mM NaHCO_3_, 1.5 mM KH_2_PO_4_, 1.4 mM MgSO_4_·7 H_2_O, 2 mM CaCl_2_·2 H_2_O, 3 mM KCl, 10 mM Glucose; osmolality = 300 mOsm) were collected using an oxygen meter (Microelectrodes Inc., OM-4) interfaced to a volume-limited microelectrode probe (Microelectrodes Inc., MI-730). Multiple trials (n = 6) were conducted in which dissolved oxygen measurements were taken from the perfusate reservoir which was directly bubbled with 95% O_2_, 5% CO_2_ carbogen (positive control) or from the perfusion chamber during trials in which the in-bore oxygenator was supplied with gases of varying oxygen content (ambient air, 95%, 60%, & 19%). In order to compare the in-bore oxygenator’s function to the external membrane oxygenator, additional measurements (n = 10) were made in the perfusion chamber while employing the external oxygenator device supplied with carbogen gas (95% O_2_, 5% CO_2_) during rig operation (2 ml/min).

### pH of Bicarbonate Buffered aCSF Perfusate

pH readings of aCSF perfusate were assessed using an Accumet Basic pH meter (Fisher Scientific, AB15) interfaced to a low-volume sample probe (Mettler Toledo, 6030-02-BNC). Measurements were taken in the perfusate well (n = 8) with the operational perfusion rig (2 ml/min) interfaced to either the external or in-bore membrane oxygenator. The aCSF reservoir was directly bubbled with carbogen gas (95% O_2_, 5% CO_2_) while the same gas mixture was supplied to both external and in-bore oxygenators. Results from the eight trials were averaged and the means of both treatment groups compared to the target physiological pH range (7.3–7.4).

### Stability Testing: Diffusion-Signal Comparisons With and Without Perfusion Over Protracted Scan Times

All imaging was performed on a 600 MHz spectrometer (Oxford) equipped with microimaging gradients (Bruker Biospin; Micro 5). Sample temperature (23 °C ± <1 °C) was measured continuously and kept constant using a feedback loop from the thermocouple to a chiller unit (Bruker, BCU II −80/60) which controls air temperature flowing through the spectrometer bore. All data was collected using a diffusion-weighted, spin echo sequence. Diffusion weighting was kept constant at b = 1200 s/mm^2^: a compromise offering sufficient sensitivity to tissue changes as well as adequate signal-to-noise ratio (400–500 typical SNR) in the available scan time. Data analysis was performed offline using a statistical toolbox (Microsoft Excel) and Matlab®. Fourteen consecutive diffusion-weighted, spin echo scans (TR/TE = 2000/11.64 ms, b = 1200 s/mm^2^, Res = 31.25 μm isotropic, duration = 1.5 h, Avg = 42) were collected (n = 3) comparing signal stability over time (21 h total). Manual shimming was employed to ensure homogeneous field conditions^28^. Shim settings were evaluated using measures of linewidth at ½ height of the water signal peak—typically ≤35 Hz—which were observed in both the presence and absence of the in-bore oxygenator: i.e. use of the device did not induce detectable field inhomogeneities. Identical imaging protocols were employed in each of two experimental groups: one series with continuous perfusion (2 ml/min) and one series with no perfusion (stable control). Raw diffusion signal readings—obtained as average signal intensity across a region of interest—from the three images taken at each of fourteen time points were averaged and plotted as a function of time to assess the within-group signal variability over the 21 h experiment. Statistical comparisons between treatment groups were conducted using equivalence testing. The range of equivalence (+/−9% mean) was determined based on total within-group signal change observed in the non perfused—i.e. stable static control group—over the timecourse of the study. Both imaging datasets for our stability testing were conducted on fixed mouse cortex (300 μm thick) to eliminate morphological sample changes as a possible cause of any diffusion signal changes observed.

### Live Slice Perfusion: Diffusion-Signal Behavior in Acute Slice Preparations Compared to Non Perfused Tissue and a Fixed, Static Control

Acute cortical slices (n = 4, 300 μm thick) from rats were isolated via vibratome in a continuously gassed (95% O_2_, 5% CO_2_), 4 °C aCSF bath before being introduced into the microperfusion rig. Samples were acclimated to 23 °C under continuous perfusion for a period of 1 h prior to imaging. Diffusion weighted images (TR/TE = 2000/11.6 ms, b = 1200 s/mm^2^, Res = 31.25 μm isotropic) were collected at long and short imaging intervals (duration = 1.5 h, Avg = 42; duration = 4 min, Avg = 2) with variations in perfusion conditions (continuous = 1.5 h scans with perfusion on for entire 21.5 h timecourse; intermittent = 1.5 h scans with perfusion off during data collection but on for 10 min perfusion intervals in between; long interval/long scan = 1.5 h scans with perfusion on during data collection and off during the 10 minute intervals between scans; long interval/short scans = 4 min scans taken with perfusion off interspersed with 1.5 h intervals with perfusion on). Nonperfused cortex data was generated with a live, acute slice placed in the rig and imaged (TR/TE = 2000/11.6 ms, b = 1200 s/mm^2^, Res = 31.25 μm isotropic, duration = 1.5 h, Avg = 42) over a 15.5 h timecourse in the absence of aCSF perfusate exchange. Similarly, stable control data was generated using a fixed slice over an 18.5 h timecourse with identical imaging parameters in the absence of perfusion. Diffusion signal intensity (arbitrary units) was recorded over a timecourse lasting 15.5 h to 21.5 h depending on experimental group.

## Results

### Device Design and Fabrication

**Figure 1:**
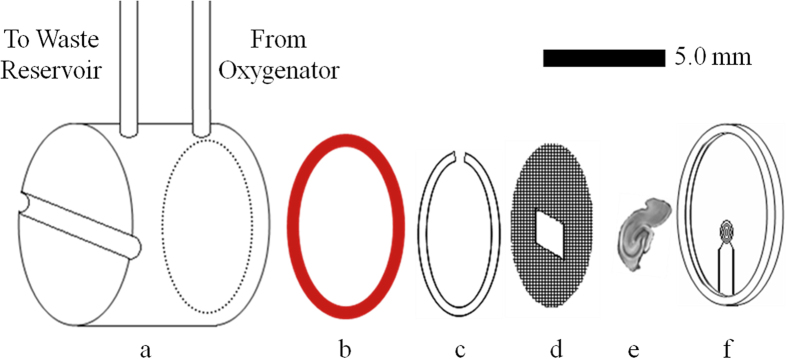
Exploded schematic detailing the individual microperfusion rig components. (**a**) Perfusate well machined from acetal rod (150 μl volume, 9.5 mm O.D., 6 mm LN, 6 mm I.D.). Open-ended side (······) is beveled 30^o^ from vertical to accommodate seating of a silicone gasket. The closed side contains a horizontal channel (2.5 mm HT × 1 mm D) in which a low-profile cable tie (Thomas & Betts, SF100-18) (not pictured) sits to serve as a nonpermanent means of sealing the perfusion chamber. Inflow and outflow lines (Cole-Parmer, S-06418-02) enter through the top of the perfusion well and are affixed in place with high peel-strength urethane applied externally. (**b**) Silicone o-ring (Amazon, ORS-009-25) which serves as the liquid-tight gasket between the perfusion well and tissue well. It is affixed to the perfusion well using aquarium-safe silicone sealant. (**c**) Nylon retention ring (5 mm O.D., 4 mm I.D., 0.5 mm wall, 300 μm thick) fashioned by hand from a flat nylon washer (Amazon, B00DHVBPOO). The internal diameter was widened to 3.97 mm using a sheet-metal punch. The thickness was reduced using a metal file. Lastly, a notch (2 mm) was cut into the ring with a scalpel to allow for compression prior to insertion into the tissue well. (**d**) Woven nylon (Amazon, CMN-0053-C) of 50 μm pore size was used in construction of the retention mesh. A circular disk (4 mm Dia.) was cut from the sheet and a window (2 mm × 1.5 mm) was cut from the center portion overlapping the coil face. This window aided tissue placement by allowing for clear viewing of the sample region in contact with the coil and prevented nylon from entering the coil’s excitation profile. (**e**) Tissue slices (hippocampus pictured) 300 μm thick are placed in direct contact with the surface coil and suspended vertically using the mesh insert and retention ring. (**f**) The four-turn micro surface-coil (500 μm Dia.) is pictured at the bottom of the 5 mm Dia. tissue well. Additional components of the coil assembly (leads, capacitors, plastic base, etc.) are not shown.

**Figure 2 f2:**
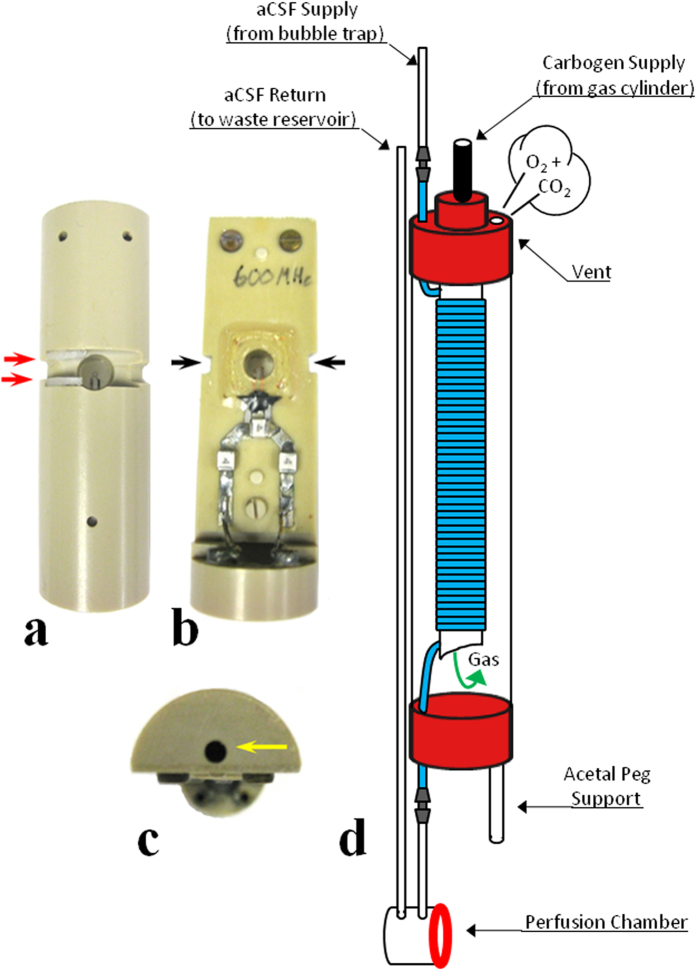
Photographs of the modified 500 μm microcoil and detailed schematic of the in-bore oxygenator. (**a**) Rear-view of the microcoil assembly illustrating a milled channel (15 mm LN × 3 mm HT × 4 mm D) flanked by nylon spacers (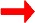, 6 mm LN × 4 mm HT × 0.5 mm W) which catch a low-profile cable tie (Thomas & Betts, SF100-18). (**b**) Front-view of the microcoil detailing bilateral groves (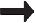, 3 mm HT × 1.5 mm D). Cutaways provided clearance for the cable tie used to reversibly seal the perfusion well. (**c**) Top-view of the coil assembly. A hole (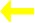
2 mm Dia. × 14 mm D) accommodates the in-bore oxygenator’s acetal support peg. (**d**) Detailed schematic of the in-bore oxygenator device (nested, open-ended 5 mm & 10 mm NMR tubes; 19 cm tall x 1 cm wide). After passing through an in-line bubble trap, aCSF perfusate enters the in-bore oxygenator through highly gas-permeable silicone tubing (HelixMark, 60-011-03). This tubing (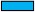) which constitutes the gas exchange membrane of the oxygenator device has been coiled tightly inside the nested NMR tubes so as to maximize the surface area over which gas exchange occurs. Carbogen gas enters through a port in the top of the 5 mm tube’s cap and passes into the gas-exchange chamber (10 mm NMR tube) through the 5 mm tube’s open bottom (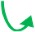). Placement of a vent at the top of the 10 mm tube ensures the carbogen passes over the coiled membrane as gas exits the oxygenator assembly. Carbogen-saturated aCSF exits through the bottom cap of the 10 mm tube and enters the perfusion chamber. The drastically reduced distance between the site of gas exchange and the site of tissue perfusion (2 cm in-bore vs. 500 cm external) accounts for the greatly improved gas retention properties observed. Artificial CSF exits the rig through a return line on its way to a waste reservoir located outside the spectrometer. Ends of the silicone membrane (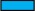) were spliced to Tygon® lines (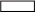) (Cole-Parmer, S-06418-02) using nylon couplers (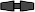) (Eldon James, C0-1AGHDPE).

**Figure 3:**
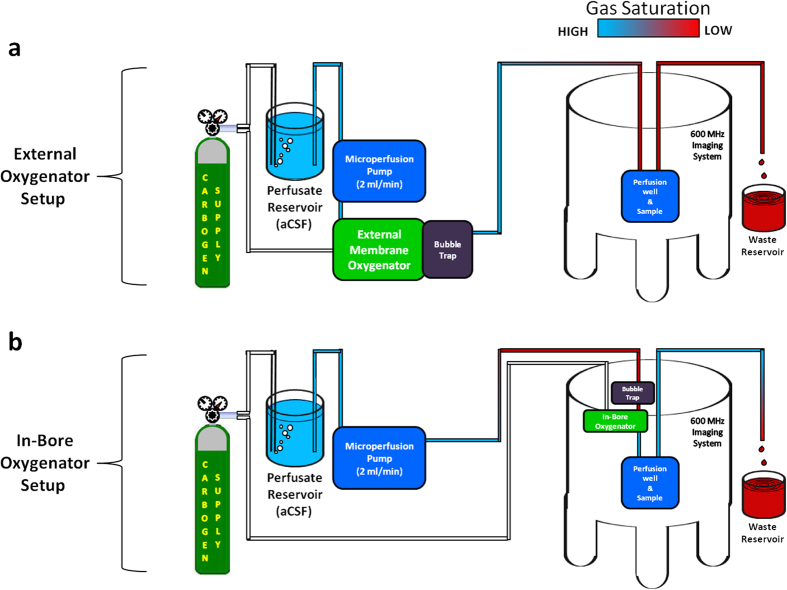
Block diagrams of the microperfusion rig in dual oxygenator configurations. (**a**) External oxygenator setup. Excessively long microperfusion lines (5 m) (Cole-Parmer, 06508-13) between the external oxygenator (45 cm wide × 60 cm tall × 30 cm deep) and perfusion chamber lead to significant degassing of our aCSF perfusate prior to encountering the tissue slice. This degassing was responsible for the low oxygen (O_2_ loss) and high pH (CO_2_ loss) conditions recorded in the perfusion chamber with the external oxygenator setup and was encountered despite our use of perfusion lines designed for high gas retention. (**b**) In-bore oxygenator setup. The membrane oxygenator (19 cm tall × 1 cm dia.) and bubble trap (9 cm tall × 1 cm dia.) portions of the microperfusion rig have been redesigned using MR-compatible materials exclusively. In addition, the dimensions of these components have been reduced such that they are now able to fit directly inside the narrow channel of the spectrometer bore (3.5 cm dia.). While significant degassing still occurs between the carbogen bubbled aCSF reservoir and imaging spectrometer, the in-bore oxygenator device drastically reduces the length of perfusion line between the terminal point of gas exchange and the tissue sample (2.5 cm) thus preserving physiologically relevant dissolved oxygen and pH values.

Detailed schematics of the micro surface-coil modification, microperfusion rig assembly, and in-bore oxygenator device are provided (Figs 1 and 2). A block diagram of the rig assembly with both the external and in-bore oxygenator attachments is also illustrated [Fig f2](Fig. 3).

### Dissolved Gas Testing in the External and In-Bore Membrane Oxygenators

**Figure 4:**
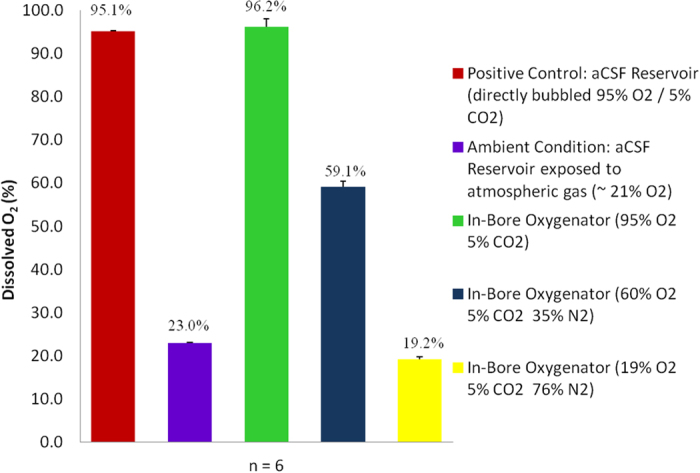
Dissolved oxygen gas (O_2_) properties of aCSF at the site of tissue perfusion using the in-bore oxygenator with gases of variable oxygen content. Three mixtures of carbogen gas (5% CO_2_ + 95%, 60%, 19% O_2_ balance N_2_) are compared to an aCSF reservoir exposed to atmospheric gas (20–22% O_2_; 23% measured) and positive control (directly bubbled 95% O_2_, 5% CO_2_ carbogen). In all three instances, measured dissolved oxygen content in the aCSF approaches 100% saturation of the concentration contained within the supplied gas mixture. Data is presented as group averages (n = 6) with positive error bars equal to the calculated standard deviation.

Dissolved oxygen content measured in aCSF at the tissue well (n = 6) as a function of gases with variable oxygen content as supplied by the in-bore oxygenator device are shown (Fig. 4). Atmospheric gas (20–22% O_2_) and three mixtures of carbogen (5% CO_2_) with varying concentrations of oxygen (95%, 60%, 19% O_2_) balance nitrogen were tested in the in-bore oxygenator. Average dissolved oxygen readings for the results of six trials were 23.0%, 96.2%, 59.1% and 19.2% for ambient air (20–22%), 95%, 60%, and 19% O_2_ gases respectively. Results are compared to positive control trials (n = 6) conducted in the perfusate reservoir during direct bubbling of 95% O_2_, 5% CO_2_ carbogen gas: 95.1% O_2_ measured. Repeat measurements indicate a 100% gas saturation effect (i.e. percent saturation at tissue well matches the % O_2_ content of supplied gas) when using the in-bore oxygenator. Conversely, when employing the external oxygenator, dissolved gas content at the site of tissue perfusion was not equivalent to the percent oxygen content of the supply gas (Table 1). In repeated testing (n = 10) average dissolved oxygen content in the aCSF at the tissue perfusion site was 43.43% O_2_ when employing a 95% O_2_ concentration supply gas. This represents a loss of 54.3% total dissolved O_2_ occurring between the perfusate reservoir (95.54% O2 positive control) and the perfusion chamber (43.43% O_2_) as measured using the external membrane oxygenator.

### pH Control in Bicarbonate Buffered aCSF

The results of multiple trials (n = 8, supply gas = 95% O_2_, 5% CO_2_) measuring pH at the site of tissue perfusion are presented (Table 1). Average pH reading in aCSF using the in-bore oxygenator device (7.32) was compared to that taken using the external oxygenator (8.13). Only readings taken when employing the in-bore oxygenator fell within a target pH range chosen for its ability to maintain physiologically relevant neural tissue metabolism (7.3–7.4). Results of pH measurements from a directly bubbled aCSF reservoir (positive control, 7.36) fell within the physiological range while those taken from an untreated (i.e. degassed) aCSF reservoir (negative control, 8.22) were more similar to readings seen when using the external oxygenator.

### Signal Stability Testing During Periods of Constant Perfusion

**Figure 5:**
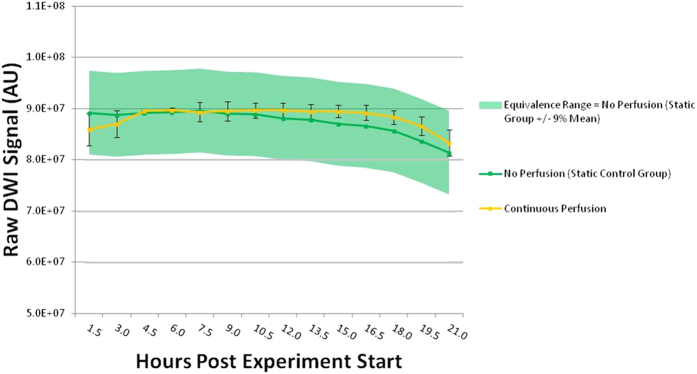
MR signal stability testing in fixed mouse cortical slices (300 μm) under conditions of constant perfusion and no perfusion. Diffusion-weighted signal (arbitrary units) over time (21 h total) in imaging series with continuous (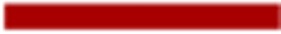: 2 ml/min) and static (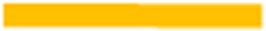: no perfusate flow) control. Average signal (n = 3) over 12 separate imaging experiments is reported. One of three measurements at the 4.5 and 6.5 h time points were excluded from the third series due to a hardware malfunction which affected collection. Statistical testing shows that the conditions for between-group equivalence (range = signal variance over time [21 h] exhibited by our static control group [ ±9%]) were met at all 12 of the time points tested. Data is presented as group averages with positive and negative error bars equal to the standard deviation. Error bars were omitted from the static control group for clarity.

Diffusion-weighted MR signal (arbitrary units) as a function of experiment time (21 h total) is reported for two experimental conditions: trials with continuous (2 ml/min) perfusion and static trials with no perfusion (control) (Fig. 5). Average signal from multiple experiments (n = 3) is calculated and graphed at 12 separate 1.5 h time points. Statistical testing for equivalence determined that the criteria for equivalence was met at all time points tested. Data presented at the 4.5 and 6.0 h time points (n = 2) contained one fewer measurement than the remaining groups due to a hardware malfunction which occurred during the collection of the third series (Figs S1 and S2) and was thus excluded from statistical testing.

### Live Slice Characterization Using the Microperfusion and In-Bore Oxygenator Apparatus

**Figure 6:**
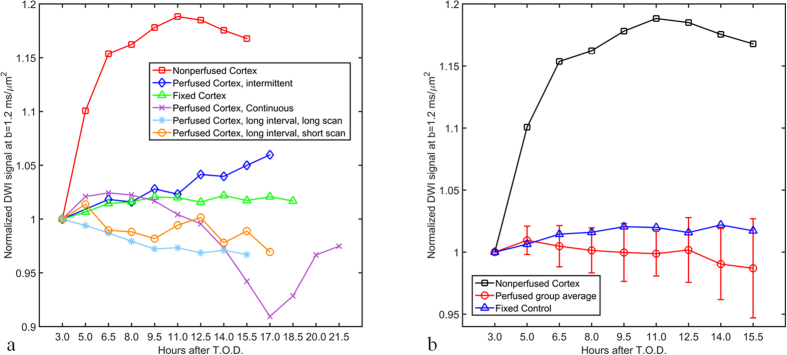
Characterization of live, acute cortical slices from rats using the microperfusion and in-bore oxygenator apparatus. Diffusion-weighted MR signal (arbitrary units) normalized to the initial measurement (3 h) as a function of experiment time (15.5 to 21.5 h) is reported. (**a**) Experiments utilizing four independent perfusion protocols are compared to fixed tissue (stable control) and non perfused, live tissue (metabolic insufficiency). Non perfused cortex exhibits an abrupt and sustained increase in diffusion signal (3 to 15.5 h) while the fixed sample remains relatively constant throughout (3 to 18.5 h). Acute cortical slices undergoing perfusion exhibit modest signal changes over time which are intermediate to those observed under the two control conditions. Perfused Trials Key (continuous = 1.5 h scans with perfusion on for entire 21.5 h timecourse; intermittent = 1.5 h scans with perfusion off during data collection but on for 10 min perfusion intervals in between; long interval/long scan = 1.5 h scans with perfusion on during data collection and off during the 10 minute intervals between scans; long interval/short scans = 4 min scans taken with perfusion off interspersed with 1.5 h intervals with perfusion on). (**b**) Upon grouping the data from perfused trials over their shared timecourse (3 to 15.5 h) these slices exhibit diffusion signal stability behavior similar to fixed-tissue controls. Diffusion-weighted MR signal (arbitrary units) normalized to the initial measurement (3 h) is reported as a function of time. Data in is reported as group means (n = 4) with positive and negative error bars equal to the standard error of the mean.

Tissue stability as indicated through the diffusion-weighted MR signal intensity was analyzed for all live slice data sets as a function of experiment time (15.5 to 21.5 h) (Fig. 6). The reported signal at each time point is the mean signal intensity over a large region-of-interest (ROI). For each sample, the same ROI was used for all time points. To allow comparison of signal stability between the perfusion strategies, we normalize each data series to the intensity at its initial measurement (3 h). In this data presentation, insufficiently perfused tissue would display increased signal intensity over time as a result of reduced diffusivity caused by ischemia. We also include the signal curve from fixed tissue (also normalized to its intensity at the initial time point) as a reference time series of a completely stable sample. Experiments utilizing four independent perfusion protocols are compared to fixed tissue (stable control) and non perfused live tissue (insufficient metabolite control). As expected, the non perfused cortex (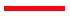, Fig. 6a) displays an abrupt and sustained increase in diffusion signal behavior. Conversely, diffusion measurements obtained from the fixed sample remain relatively constant throughout the 18.5 h timecourse (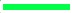). Perfused, live cortical slices (Fig. 6a) exhibit varying degrees of diffusion signal change with the most prominent seen in the continuously perfused cortex sample (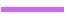) following the 14 h time point. In Fig. 6a, the remaining perfusion regimes are: intermittent perfusion (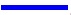), long perfusion intervals followed by short scans (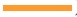), and long perfusion intervals followed by long scans (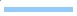). Please refer to the method section on Live Slice Perfusion for interval lengths.

Figure 6b shows all these perfusion strategies grouped together and compared to the signal time course of a non-perfused acute slice and a fixed sample. This graph is limited to the timecourse inclusive to all measured conditions (3.0 h to 15.5 h; Fig. 5b). The perfused cortical slices exhibit stable diffusion signal characteristics akin to fixed-tissue controls.

## Discussion and Conclusions

In the present study, we describe a newly designed and fabricated microperfusion system and oxygenator device for use in explant studies conducted in ultra-high-magnetic field environments. These components were designed specifically to conform to the intense spatial and material limitations imposed by high-field MR imaging equipment used in cellular studies and thus possess particular features not available in any existing commercial microperfusion equipment. Importantly, the rig materials are all compatible with the electromagnetic environment (static and time-varying fields) of the MR experiment so that they are both safe to handle and do not interfere with measurement quality (e.g. by deteriorating the main field homogeneity). What’s more, our in-bore oxygenator device exhibited 100% dissolved oxygen (O_2_) saturation characteristics tested using multiple concentrations of oxygen in pre-mixed carbogen supply gas (Fig. 4). These results indicate a virtual 0% O_2_ loss in the oxygen gas saturation of our aCSF when using the in-bore oxygenator and microperfusion rig. In addition, they demonstrate that O_2_ saturation at the site of tissue perfusion can be precisely controlled by regulating its concentration in the mixture of supply gas. This feature increases operator control by allowing researchers to tailor experimental conditions based on tissue-specific—and even activity dependent—differences in metabolic requirements^29^,^30^,^31^,^32^. Such precision and control at the location of tissue perfusion—in this case, inside the bore of an imaging spectrometer—are indispensible to creating well constructed, reproducible scientific studies with *ex vivo* sample preparations. Our finding that more than half (54.3%) of our aCSF’s dissolved O_2_ content was lost during travel from an external membrane oxygenator to the tissue well (5 m) highlights the need for additional MR-specific hardware development. That such results were obtained even when employing high-retention, low gas permeability perfusion lines (Cole-Parmer, 06508-13) suggests that simple engineering solutions retrofitted to preexisting perfusion equipment will not be adequate to achieve appropriate experimental conditions in *ex vivo* MR microscopy studies.

Although the current study did not include direct quantification of dissolved CO_2_ gas as was performed with oxygen, pH readings in our bicarbonate buffered aCSF served as a sensitive measure of the gas’ effects. This is due to the role supplied CO_2_ plays in formation of carbonate and bicarbonate ions—and thus free protons—through a carbonic acid intermediate as part of the bicarbonate buffering system:


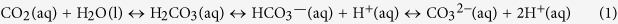


The chemical constituents described are the same utilized naturally for the purpose of regulating physiological pH in mammals via respiratory and excretory metabolic processes^33^,^34^. When supplied to perfusate or culture media containing a bicarbonate buffer, CO_2_ acts to stabilize pH within a physiologically acceptable range for mammalian neural tissues: 7.3–7.4^35^,^36^. This is why CO_2_ is used ubiquitously in carbogen gas mixtures (95% O_2_, 5% CO_2_) supplied to bicarbonate-buffered tissue culture systems. Addition of CO_2_ into such a system results in an increase in carbonic acid and thus an increase in the bicarbonate and hydrogen ions this compound dissociates into ultimately resulting in a lowering of pH. Conversely, if conditions in the media become too acidic, excess hydrogen ions will be scavenged by bicarbonate and become incorporated in water molecules upon the release of CO_2_ resulting in an increase of pH. Maintaining physiologically relevant pH conditions in a bicarbonate buffered media thus depends on a constant supply of CO_2_ gas. The concentration of CO_2_ present in carbogen mixtures used in the current study (5%) is an industrial standard known to be sufficient to maintain these conditions; however, when this was used as a supply gas in conjunction with the external membrane oxygenator, pH measured at the site of tissue perfusion was 8.13: excessively alkaline compared to our target pH range of 7.3–7.4. These data were observed despite target pH conditions being maintained in the aCSF reservoir used to supply the rig with perfusate (Table 1). Our pH measurements taken using the external oxygenator coupled with our knowledge of the bicarbonate buffer chemistry described above suggested that an insufficient supply of bicarbonate ion—and thus free protons—was being maintained in our aCSF at the site of tissue perfusion. Since this problem was not present in our aCSF upstream of the tissue well, we were able to conclude that the unintended shift in pH developed as our perfusate traversed the length of the perfusion rig: specifically the 5 m of perfusion lines connecting the external membrane oxygenator to our tissue perfusion chamber. The most likely cause for the observed shift was thus determined to be a decrease in pCO_2_ occurring over the protracted distance of perfusion lines required to reach the center of the imaging spectrometer. Results of our O_2_ tests using the external oxygenator described above (Table 1) later confirmed that an inordinate amount of dissolved gas was escaping through our perfusion lines by diffusive processes.

While a variety of buffers are available—most notably the Good’s buffers employed in the majority of biological research^37^—non-bicarbonate buffered perfusate systems have been shown to alter the resting membrane potential of hippocampal neurons^38^. Thus, while physiologically relevant pH conditions may have been obtained more easily by replacing the sodium bicarbonate with an alternative buffering agent, certain considerations—including an eye towards future functional studies—prevented us from employing such a solution. What’s more, while changing buffers may have allowed us to achieve a physiologically relevant pH when employing the external oxygenator, issues with the loss of dissolved oxygen gas would have remained.

Upon integration of the in-bore membrane oxygenator into our microperfusion rig, pH readings in the tissue well were regulated to within the target pH range (Table 1). The distance between the final site of perfusate gassing and the tissue sample was reduced by a factor of 250 (5 m vs. 2 cm) after exchanging oxygenator devices (Fig. 3). Such a reduction in the length of perfusion lines between sites of gas exchange and tissue perfusion prevented the loss of dissolved CO_2_ by decreasing both the overall surface area and time over which these diffusive losses could occur.

The vast majority of *ex vivo* MR microscopy studies to date have employed discontinuous perfusion as a part of their imaging protocol so as to eliminate flow artifacts arising from moving perfusate^39^,^40^,^41^,^42^,^43^,^44^,^45^,^46^. While control experiments in these studies have reported MR signal stability using intermittent perfusion methods, metabolic studies have shown that tissue explants benefit physiologically from the continuous turnover of perfusate^47^,^48^. Such results are not surprising given the perfusate’s role in both delivering vital nutrients and removing the toxic byproducts of metabolism. In the current study, due to the micro surface-coil’s shallow penetration depth and its spatial separation from the perfusate due to the tissue partition (300 μm, Fig. 1), we hypothesized that perfusion could be maintained during image acquisition without generating flow artifacts. To test this, we compared diffusion acquisitions of fixed mouse cortex over time (21 h) under conditions of both continuous perfusion and static, motionless perfusate (Fig. 5). In similar diffusion-weighted scans of perfused, acute hippocampal slices performed within our research group, signal stability was demonstrated over an 8 h time interval using an intermittent perfusion protocol which provided 5 min perfusion at a rate of 2 ml/min for every 1.5 h of scan time^49^. The authors reported an approximately 8% variation in raw diffusion signal over the 8 h interval for their stable, perfused tissue group. This value is quite close to the 9% raw diffusion signal variability reported in the current study for our static control group (Fig. 5). Given the differences between the two studies—particularly the use of acute rather than fixed slices—the strikingly similar diffusion signal variation observed is more likely a result of stability limitations inherent to the imaging hardware employed rather than changes occurring within the tissue sample.

In the case of our live tissue experiment performed on acute cortical slices, diffusion signal stability was maintained for a period of 15.5 h following time of death (T.O.D.) and subsequent sample preparation prior to imaging (Fig. 6). Slice thickness (300 μm) and running temperature 23^o^ C were selected to achieve sufficient oxygen tension throughout the sample while perfusate conditions (95% dissolved O_2_, 2 ml/min flow rate) ensured efficient metabolite delivery and waste removal^50^,^51^. Conversely, the early increase in diffusion signal intensity observed in the non-perfused, live slice was concurrent with cellular edema brought about by ATP depletion-related failure of ionic pumps within the plasma membranes of the slice’s cells^52^,^53^. Hypoxia-induced tissue swelling is the physical mechanism responsible for the decrease in diffusivity within the slice which manifests as an increase in diffusion-weighted MRI signal. Samples perfused using the in-bore oxygenator exhibited far more diffusion signal stability than the metabolite-compromised control, as their diffusion signal change over time was similar to that observed in the fixed tissue specimen. As may have been expected, between-group variability increased as a function of time in the averaged data from our four perfused samples which can be appreciated by the increasing amplitude of the error measured over time (Fig. 6b). Interestingly though, the continuously perfused sample exhibited by far the most change in diffusion signal over time of the four perfused slices despite the assumption that this tissue was exposed to the most favorable metabolic conditions (Fig. 6a). It should be noted that these changes occurred after the 14 h timepoint: later than the 6 to 12 h timecourse over which acute slices are viable for electrophysiological recordings and much later than the 4 h limit after which compromised, argyrophilic neurons have been observed under similar physiological maintenance^54^. Such affects on slice health have been linked to exposure to lipopolysaccharides and lipopeptides present in the cell walls of bacteria^55^. These bacteria are present in perfusion media and undergo logarithmic increases in population density which begins between the 6th and 12th h of incubation. Fortunately, engineering solutions have already been developed which prolong the rate of proliferation of bacteria in aCSF perfusate and subsequently the time period over which acute slice preparations remain functionally viable. Using temperature suppression and ultra violet (UV) light filtration, researchers at the University of Western Sydney have fabricated a specialized perfusion system which is capable of preserving the electrophysiological characteristics of acute slices for periods of 24 to 36 h^56^. The demonstrated success of such techniques has immense implications for the collection of MR microscopy images which typically take hours to complete. Incorporation of a UV filter device upstream of the in-bore oxygenator in the current setup—perhaps integrated into the bubble trap mechanism—has the potential to double or triple the viability period in tissue explant studies.

It is our hope that the current rig design described and tested here will be employed in cellular resolution MR studies of living tissue explants as well as aid in the future development of MR-compatible perfusion devices.

## Additional Information

**How to cite this article**: Flint, J. J. *et al.* A Microperfusion and In-Bore Oxygenator System Designed for Magnetic Resonance Microscopy Studies on Living Tissue Explants. *Sci. Rep.* **5**, 18095; doi: 10.1038/srep18095 (2015).

## Supplementary Material

Supplementary Information

## Figures and Tables

**Table 1:** Comparison of the oxygen gas (O_2_) saturation and pH characteristics in aCSF perfusate using the external oxygenator and in-bore oxygenator systems.

	pH	Dissolved O_2_
aCSF Reservoir Negative Control (Equilibrated to Atmosphere)	8.22 ± 0.016	23.02 ± 0.08%
aCSF Reservoir Positive Control (direct bubbled 95% O_2_, 5% CO_2_)	7.36 ± 0.018	95.54 ± 0.97%
External Membrane Oxygenator	8.13 ± 0.085	43.43 ± 3.52%
In-Bore Membrane Oxygenator	7.32 ± 0.019	96.18 ± 1.87%

Measurements for the external and in-bore oxygenator configurations were taken in the perfusion chamber. When employing the external oxygenator, loss of dissolved CO_2_ (degassing) from perfusion lines resulted in an excessively alkaline aCSF mixture at the site of tissue perfusion. Similar pH conditions were closely replicated in an untreated aCSF reservoir left exposed to atmospheric air (negative control). Use of the in-bore oxygenator restored CO_2_ levels in the bicarbonate-buffered aCSF perfusate as evidenced by a return to physiologically relevant pH values. Compared to positive control measurements, aCSF gassed by the external oxygenator exhibited a 54.3% drop in total dissolved oxygen content between the oxygenator and perfusion chamber. When employing the in-bore oxygenator, no measurable loss of dissolved oxygen was noted. pH testing (n = 8), dissolved O_2_ testing (n = 10 [control & external], n = 6 [in-bore]). Values are reported as group averages ± standard deviation.
