# Supplementary material for: A Microperfusion and In-Bore Oxygenator System Designed for Magnetic Resonance Microscopy Studies on Living Tissue Explants
Source: Sci Rep. 2015 Dec 15;5:18095. doi: 10.1038/srep18095 (PMC4678305; doi:10.1038/srep18095)
Supplement: Supplementary Information [file srep18095-s1.pdf]

A Microperfusion and In-Bore Oxygenator System Designed for Magnetic Resonance Microscopy Studies on Living Tissue Explants.

Jeremy J. Flint[1,2*], Kannan Menon[2,3], Brian Hansen[4], John Forder[5], Stephen J. Blackband[1,2,6]

[1] Department of Neuroscience, University of Florida, Gainesville, Florida, United States of America

[2] McKnight Brain Institute, University of Florida, Gainesville, Florida, United States of America

[3] Department of Biomedical Engineering, University of Florida, Gainesville, Florida, United States of America

[4] Center for Functionally Integrative Neuroscience, Aarhus University, Aarhus, Denmark

[5] Department of Radiology, University of Florida, Gainesville, Florida, United States of America

[6] National High Magnetic Field Laboratory, Florida State University, Tallahassee, Florida, United States of America

[*] Corresponding author

E-mail:	Dr. Jeremy Flint	jflint@mbi.ufl.edu
	Mr. Kannan Menon	kannan.menon89@ufl.edu
	Dr. Brian Hansen	brian@cfin.au.dk
	Dr. John Forder	jforder@ufl.com
	Dr. Stephen Blackband	sblackba@ufl.edu

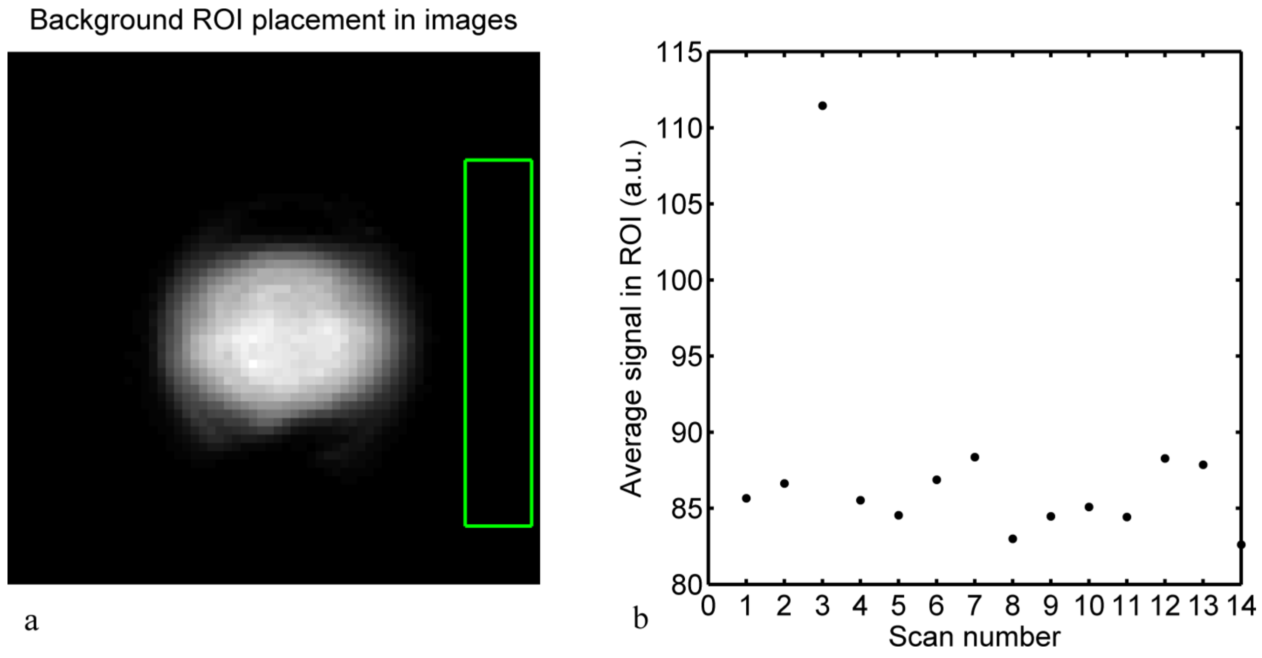

Figure S1. Increased noise readings in the 4.5 h time point (scan 3) of fixed slice stability experiment (1 of 3 trials). (a) Representative diffusion image showing the noise ROI ( ) calculated for each of 14 scans in the 21 h series. (b) Signal intensity graph of the noise region measured at each time point. Note the sharp increase in noise observed during the third scan.

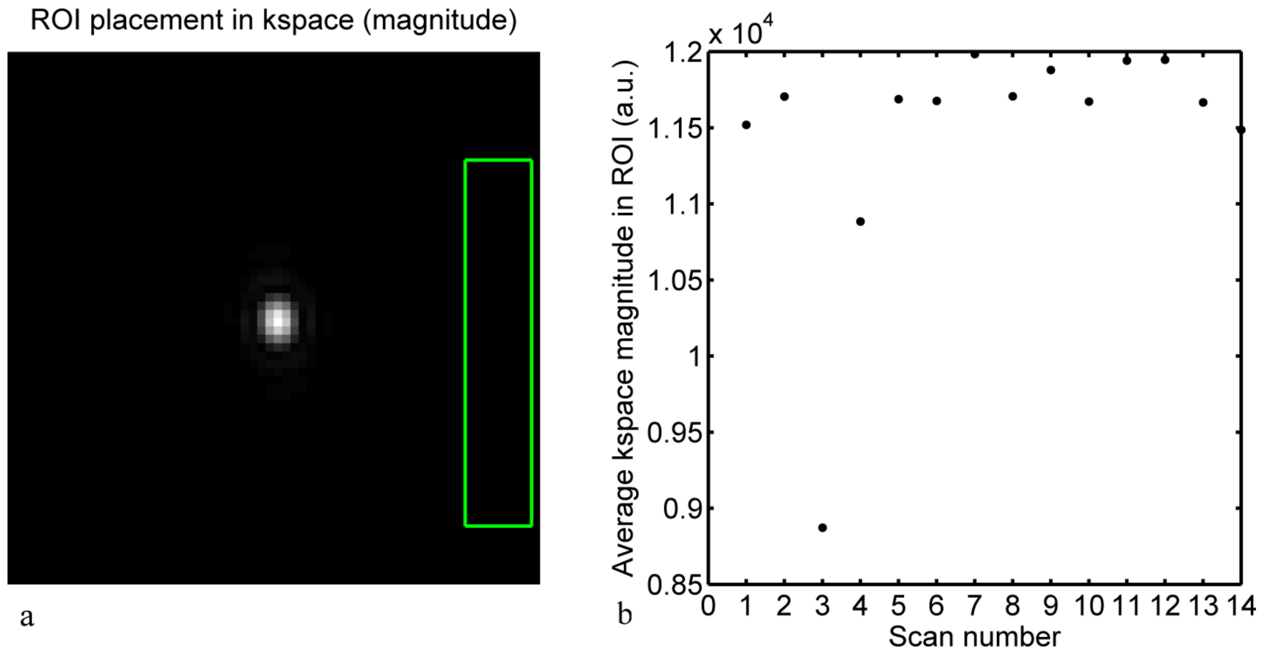

Figure S2. Decreased k-space intensity in the 4.5 h and 6.0 h time points (scans 3 and 4) of fixed slice stability experiment (1 of 3 trials). (a) Representative k-space image showing the ROI ( ) calculated for each of 14 scans in the 21 h series. (b) Signal intensity graph of the ROI measured at each time point. Note the sharp decrease in magnitude observed during the third and persisting into the fourth scans.
